# Conflicts of Interests: Declarations for All

**DOI:** 10.1289/ehp.112-a980

**Published:** 2004-12

**Authors:** Daniel W. Nebert

**Affiliations:** Department of Environmental Health, University of Cincinnati Medical Center, Cincinnati, Ohio, E-mail: dan.nebert@uc.edu

Concerning your editorial, “Embracing Scrutiny,” in the October issue of *EHP* [Environ Health Perspect 112:A788 (2004)], the need for full disclosure of all potential conflicts of interest by all coauthors contributing to a publication in *EHP* is commendable and obviously needed. Might I take this one step further and suggest that all reviewers of *EHP* manuscripts be required to sign a form listing all of their potential conflicts of interest.

